# Alloreactive Natural Killer Cells for the Treatment of Acute Myeloid Leukemia: From Stem Cell Transplantation to Adoptive Immunotherapy

**DOI:** 10.3389/fimmu.2015.00479

**Published:** 2015-10-15

**Authors:** Loredana Ruggeri, Sarah Parisi, Elena Urbani, Antonio Curti

**Affiliations:** ^1^Department of Medicine, Division of Hematology and Clinical Immunology, Ospedale Santa Maria della Misericordia, University of Perugia, Perugia, Italy; ^2^Department of Experimental, Diagnostic and Specialty Medicine, Institute of Hematology “L. and A. Seràgnoli”, S. Orsola-Malpighi Hospital, University of Bologna, Bologna, Italy

**Keywords:** natural killer cells, acute myeloid leukemia, stem cell transplantation, immunotherapy, alloreactivity

## Abstract

Natural killer (NK) cells express activating and inhibitory receptors, which recognize MHC class-I alleles, termed “Killer cell Immunoglobulin-like Receptors” (KIRs). Preclinical and clinical data from haploidentical T-cell-depleted stem cell transplantation have demonstrated that alloreactive KIR-L mismatched NK cells play a major role as effectors against acute myeloid leukemia (AML). Outside the transplantation setting, several reports have proven the safety and feasibility of NK cell infusion in AML patients and, in some cases, provided evidence that transferred NK cells are functionally alloreactive and may have a role in disease control. The aim of the present work is to briefly summarize the most recent advances in the field by moving from the first preclinical and clinical demonstration of donor NK alloreactivity in the transplantation setting to the most recent attempts at exploiting the use of alloreactive NK cell infusion as a means of adoptive immunotherapy against AML. Altogether, these data highlight the pivotal role of NK cells for the development of novel immunological approaches in the clinical management of AML.

## Introduction

The clinical management of acute myeloid leukemia (AML) relies on aggressive chemotherapy, followed by allogeneic stem cell transplantation (SCT). Although the post-chemotherapy complete remission (CR) rate ranges from 60 to 85% in younger patients, the disease relapse is still very high, thus reducing overall survival (OS) to 40%. Of note, the prognosis of elderly patients is particularly poor with an OS of about 10%. Such a particular dismal clinical outcome is due to an increase in unfavorable biological features, which reduces the CR rate and, whenever CR is obtained, to the inability to undergo post-CR consolidation programs, including SCT, due to co-morbidities. In the attempt to improve AML clinical outcome, novel regimens and targeted therapies have been proposed in the last few years but the clinical results have proven limited. In particular, a minimal residual disease (MRD), resistant to further treatments, often persists after induction chemotherapy. In that context, the use of an immunological approach to target MRD may significantly impact on the eradication of disease. The proof-of-principle of the capacity of immune cells to eradicate MRD derives from the results of allogeneic SCT, which clearly represents an option for relapse prevention. In particular, the critical role of natural killer (NK) cells as key players in AML prevention and eradication has been clearly established, especially in the context of haploidentical SCT. However, the SCT approach has important limitations and is not applicable to all patients. For these reasons, it is conceivable to exploit the anti-leukemia potential of NK cells outside the transplantation setting as adoptive immunotherapy.

## NK Cells: Biological Pills

**Figure 1:**
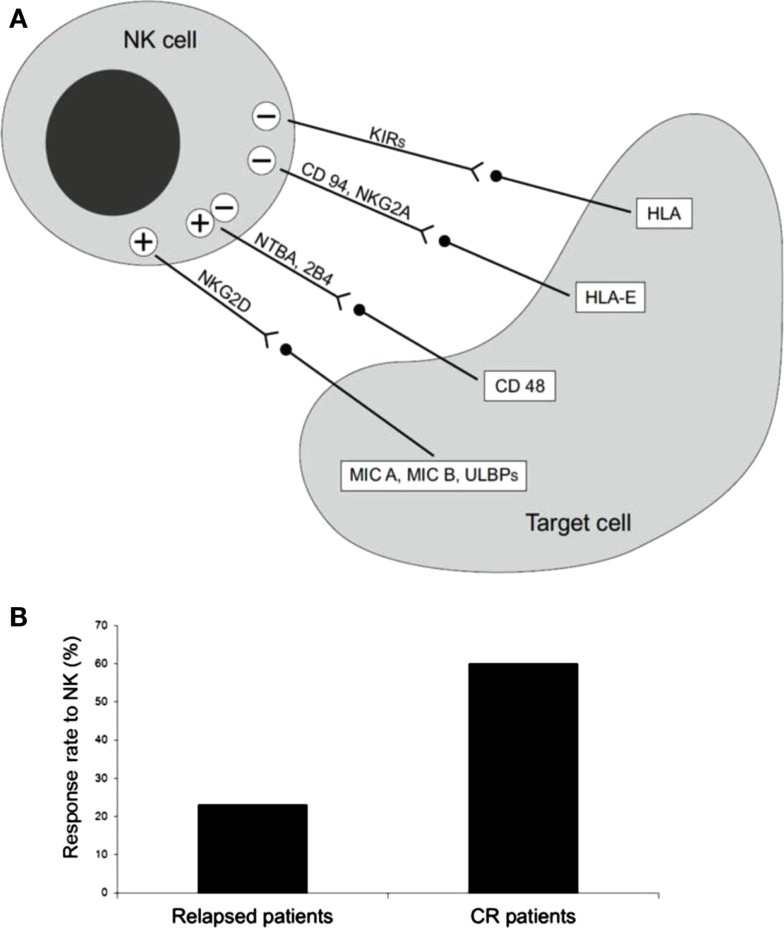
**(A)** Receptors and ligand involved in NK cell-mediated cytotoxicity. **(B)** Percentage of long-term CR patients after NK cell infusion. Thirteen AML patients, five with active disease, two in molecular relapse, and six in morphological complete remission (CR) were treated with alloreactive NK cells, after fludarabine/cyclophosphamide immunosuppressive chemotherapy. Only one of the five patients with active disease achieved transient CR, whereas the other four patients had no clinical benefit. On the contrary, five out of eight patients showed a response, which in some cases was long-lasting CR [adapted from Curti et al. (5)].

Within allogeneic SCT, donor lymphocytes recognize and destroy the recipient’s residual leukemic cells. The demonstration that this process, known as graft versus leukemia (GvL) effect, plays a major role in the therapeutic effect of SCT has led to the development of novel strategies of adoptive immunotherapy before and after SCT (1). Although most of the data refer to allogeneic T cells as GvL mediators, it is known that other subsets of circulating lymphocytes, such as NK cells, may significantly act as effector cells against leukemia in the post-transplantation setting. NK cells are defined by the expression of CD56 and CD16 and by the absence of the T-cell marker, CD3. NK cells are involved in the innate immune response and in cancer immunosurveillance, where they kill transformed tumors in a major histocompatibility complex (MHC)-unrestricted manner (2). NK cells originate from the bone marrow (BM) and then home to secondary lymphoid tissues. They account for 10–15% of peripheral blood lymphocytes and their activity depends on the expression on their surface of several activating and inhibitory receptors that recognize MHC class-I molecules (Figure 1A); the most notable inhibitory receptors are named killer cell immunoglobulin-like receptors (KIRs), which recognize allotypic determinants within certain groups of HLA class-I alleles. The lack of expression of the specific HLA class-I allele by allogeneic target cells allows KIRs to sense the absence of the self class-I KIR-ligand (KIR-L), thus mediating NK alloreactivity. Indeed, the engagement of these NK cell receptors results in stimulation or inhibition of NK cell effector function. KIR genes are closely packed in the leukocyte receptor complex on chromosome 19q13.4 and are inherited as haplotypes. Two distinct KIR haplotypes have been identified. Group A haplotypes encode inhibitory KIRs and have a fixed gene content, whereas group B haplotypes are variable in number and combination. NK cells become functionally competent only after they encounter self-HLA molecules during a process named licensing or NK cell education. About 10–20% of NK cells remain unlicensed and are hypo-responsive. Recently, other inhibitory receptors on NK cells have been identified, such as CD94/NKG2A receptors, that recognize a non-classic MHC class-I molecule (HLA-E). CD94/NKG2A continuously recycles from the cell surface through endosomal compartments, thus facilitating its inhibitory capacity (3). NK cells can also express activating forms of KIRs and the activating receptor CD94/NKG2C that interact with the same HLA molecules as their inhibitory counterparts. Other activating receptors include natural cytotoxicity receptors (NKp46, NKp30, NKp44), DNAM-1 that interacts with CD112 and CD155, and NKG2D, which recognizes ligands, up-regulated during cellular stress, such as tumor transformation and viral infections (4). In addition, CD16 (FcgammaRIIIA) triggers antibody-dependent cellular cytotoxicity on opsonized target cells, including tumor cells. Integrins also play a central role in mediating adhesion to target cells and degranulation (4).

## NK Cell Alloreactivity in Allogeneic SCT

### Haploidentical hematopoietic SCT

Although autologous NK cell activity is usually impaired in cancer patients (6), allogeneic alloreactive NK cells from healthy donors have been shown to exert important effector cell function and could be safely infused in cancer patients without side effects (7). In particular, within the setting of allogeneic SCT, the KIR-L incompatibility between donor and recipient in the GvL direction has been demonstrated to enhance the anti-cancer efficacy of NK cells, thus providing a novel and successful platform for anti-tumor immunotherapy. In the setting of T-cell-depleted haploidentical KIR-L mismatched SCT, we demonstrated that NK cell alloreactivity mediates a powerful and protective GvL effect, which is dissociated from Graft versus Host Disease (GvHD) (8). Indeed, early after T-cell-depleted transplantation, the reconstituted NK cells represent the predominant lymphoid cell population and the hematopoietic stem cells gave origin to an NK cell repertoire, which is identical to the donor one (8–10). Of note, the GvL activity of donor NK cells is triggered when the donor’s KIRs and the recipient’s HLA class-I molecules are incompatible, and consequently when inhibitory signals in the recipient are lacking. Preliminary studies in preclinical models demonstrated that human alloreactive NK clones, infused into NOD/SCID mice, previously engrafted with human AML cells, are capable of clearing leukemia cells and improving survival. These preclinical data on the impact of donor NK cell alloreactivity were confirmed in a series of more than 112 AML patients transplanted with haploidentical donors. Patients were divided in two subgroups, according to KIR-L incompatibility in the GvH direction. In the group with KIR-L incompatibility a significantly reduced relapse rate was observed. Moreover, 5-year event-free survival was 5% in contrast to 60% observed in the group without KIR-L incompatibility, thus demonstrating that KIR-L incompatibility is the only independent predictive factor for survival in AML patients. Furthermore, alloreactive mismatched NK cells facilitated hematopoietic engraftment after infusion of haploidentical stem cells and inhibited GvHD by killing host antigen-presenting cells (9). Of note, in our adult cohort of transplanted patients, NK cell alloreactivity was not efficient in preventing disease relapse of acute lymphoblastic leukemia (ALL). In contrast, in a group of pediatric leukemia patients, including ALL, undergoing haploidentical SCT, Pende et al. showed the beneficial role of activating KIRs and donor NK cell alloreactivity (11). Other clinical studies support the protective role of alloreactive NK cells in the haploidentical SCT setting. Stern et al. showed the results of a phase II multicenter study in which purified NK cells were administered pre-emptively in recipients of T-cell-depleted haploidentical SCT. Sixteen young patients with high-risk leukemia or highly malignant solid tumors were included in this protocol and received NK-donor lymphocyte infusions (DLI) on days 40 and 100 after transplantation. This study demonstrated the feasibility of the procedure. However, as a consequence of contaminating T cells in NK-DLI cell preparation, the trial showed a high incidence of acute GvHD, whereas the anti-leukemic activity appeared to be very limited (12). The use of allogeneic donor NK cells instead of autologous NK cells for cancer therapy has been recently reported. Several groups have explored allogeneic NK cells for the treatment of relapse following HLA-haploidentical SCT. Interestingly, GvHD did not occur. Some groups have infused activated, expanded donor NK cells in patients early after allogeneic SCT. Table 1 summarizes clinical trials with allogeneic NK cells as therapeutics. In these studies, clinical responses were observed in some patients and overall rates of relapse were reduced. For patients lacking an HLA-identical donor and for those with progressive disease, the use of HLA-haploidentical family donors is now increasingly considered to be a suitable alternative. However, many different protocols of T-cell-repleted haploidentical transplantation are ongoing and few data are available on the role of NK alloreactive donors in the presence of T cells in the graft and/or when GvHD prophylaxis is administered. The results of these studies are highly warranted to better elucidate the possible competition of NK and T cells and the role of immune suppressive drugs on NK cells *in vivo*.

**Table 1:** **Clinical trials with expanded allogeneic NK cells in haploidentical SCT**.

Diseases	Phase of trials	Cells	Combined therapy	Institute
High-risk solid tumors	Ongoing phase 2	*Ex vivo* expanded NK cells	Haploidentical HSCT, RIC and IL-2	Samsung Medical Center, Korea
Hematological malignancies	Ongoing	Phase-1 IL-2-activated NK cells	Haploidentical HSCT and RIC	Institut Paoli-Calmette, France
Leukemia and myeloproliferative disease	Ongoing phase 1/2	Haploidentical HSCT, TBI and chemotherapy	*Ex vivo* expanded NK cells	M.D. Anderson Cancer Center, USA
ALL	Ongoing phase 2	K562-mb 15–41 BBL and IL-2 stimulated NK cells	Haploidentical HSCT and chemotherapy	National University Health System, Singapore
AML and ALL	Ongoing phase 1/2	*Ex vivo* expanded	NK-cells haploidentical HSCT	Asan Medical Center, Korea
Relapsed/refractory pediatric acute leukemia	Ongoing phase 2	Activated and expanded NK cells	Haploidentical HSCT and salvage chemotherapy	Hospital Universitario La Paz, Spain
Myelodisplastic syndrome and leukemia	Completed phase 1/2	IL-2-activated NK cells	Haploidentical HSCT, chemotherapy and IL-2	M.D. Anderson Cancer Center, USA

### Unrelated and matched SCT

Unlike haploidentical SCT, the role of NK cell alloreactivity in the field of unrelated SCT is controversial, even though several studies have already investigated this setting (Table 2). Some years ago Giebel et al. conducted a study involving 130 patients with hematological malignancies who underwent allogeneic SCT and received Cyclosporine, ATG and short-term methotrexate as GvHD prophylaxis. With a median follow-up of 4.5 years, the OS was 87% in patients with a KIR mismatch in the donor direction versus 48% in non-KIR-mismatched patients; disease-free survival (DFS) was 87% in the first group compared with 39% in the second one. Transplant-related mortality was 6% in the KIR-mismatched patients and 40% in non-mismatched patients (13). These results were not confirmed in studies published by other centers (14, 15), which showed a detrimental effect of KIR-L incompatibility, correlated with HLA mismatching. These controversial data demonstrated that the role of NK cells remains unclear in the setting of unrelated SCT. Several factors, such as post-transplantation immunosuppressive therapies, T-cell depletion, different stem cell sources and doses, may impact in this patient setting (13–15). In a group of donor-recipient pairs missing an inhibitory KIR-L, a beneficial role of alloreactive NK cells, transiently and randomly originated from donor stem cells, was observed (16). These cells expressed the inhibitory single KIR receptor that could not be blocked by the host cells. However, these alloreactive NK cells were not functional, thus corroborating the notion that NK cells must be educated and consequently armed by the presence of the appropriate inhibitory KIR-L (17). New data have been provided on the possible role of activating KIRs which are present on KIR B haplotypes. Cooley et al. showed that B haplotype, which is present in 60% of donors, is fundamental in preventing relapse while NK cell alloreactivity does not influence the outcome of a very large cohort of unrelated transplants (18). However, in the setting of T-cell-depleted haploidentical transplants, the presence of KIR B haplotypes is associated with reduced infection-related mortality in the group of patients transplanted from NK alloreactive donors without any impact on relapse (19).

**Table 2:** **The most relevant papers reporting the impact of KIR-L mismatch in unrelated SCT**.

Authors	Survival	TRM	Relapse	GvHD	ATG
Davies et al. (14)	↓	Not assessed	→	↑^a^,^b^	No
Giebel et al. (13)	↑	↓	↓^a^	↓^a^,^c^	Yes
Bornhäuser et al. (15)	→	→	↑	→	Yes
Malmberg et al. (17)	↓	↑	→	→	Yes

*^a^Trend, *P*-value between 0.05 and 0.09*.

*^b^GvHD grade II–IV*.

*^c^GvHD grade III–IV*.

## Alloreactive NK Cells as Adoptive Immunotherapy

Natural killer cells have already been used as a means of adoptive immunotherapy beside the SCT setting (16, 20). These studies reported on the trafficking and body distribution of infused NK cells. Based on these preliminary data, NK cell selection for immunotherapy has recently been developed at clinical level (21). In 2005, Miller et al. published the results of a seminal study in which up to 1.5 × 10^7^/haploidentical NK cells/kg were safely infused in AML and cancer patients following Fludarabine/Cyclophosphamide (Flu/Cy) immunosuppressive chemotherapy; in this study some clinical responses without GvHD were observed. Circulating haploidentical NK cells were found up to 28 days after infusion, especially when exogenous interleukin (IL)-2 was given. *In vivo* expansion of NK cells was correlated with a high IL-15 serum concentration. In particular, 19 poor risk AML patients, together with 10 metastatic melanoma patients and 13 metastatic renal cell carcinoma patients received a cell population enriched in NK cells. Five out of 19 AML patients achieved CR, NK cell adoptive immunotherapy was well tolerated and no hematological toxicity was recorded. The maximum tolerated dose of NK cells was not achieved and GvHD was not observed despite the relatively high number of infused haploidentical T cells. However, it should be noted that NK cells were only partially purified after a single round of depletion of CD3^+^ cells which resulted in less than a 2 log reduction of T cells (21). A group of 10 low-risk pediatric AML patients were treated with haploidentical KIR–HLA mismatched NK infusion. All patients were alive at the 2-year follow-up. As compared to the adult trial by Miller’s group, the median number of infused NK cells was significantly higher and NK cells were processed to obtain a highly purified cell population (22). We reported the results of a trial of NK cell-based adoptive immunotherapy in 13 AML patients, 5 with active disease, 2 in molecular relapse, and 6 in morphological CR. The median age was 62 years (range 53–73). Highly purified CD56^+^CD3^-^ NK cells from haploidentical KIR-ligand mismatched donors were infused after fludarabine/cyclophosphamide immunosuppressive chemotherapy. No signs of GvHD and/or NK cell-related toxicity were reported. As expected, patients with active disease had no clinical benefit. Interestingly, both patients in early molecular relapse achieved CR and three patients in CR were disease-free after a follow-up of 34, 32, and 18 months of follow up (Figure 1B). Infused NK cells were detected in the peripheral blood of all evaluable patients and in the BM in some cases. Importantly, infused NK cells were demonstrated *ex vivo* to be alloreactive by killing *in vitro* the recipient’s cells, including leukemia (5). Several biological factors, both of recipient and donor origin, may be implicated in the therapeutic effect of NK cells after infusion into AML patients. Miller and collaborators recently reported on the critical impact of some components of the recipient immune response on the anti-leukemia activity of infused NK cells (23). In particular, they reported NK cell expansion correlates with the post-chemotherapy serum concentrations of some cytokines, such as IL-15 and IL-35, and the number of T regulatory cells (T_regs_) critically influences the capacity of infused NK cells to expand and to kill AML cells. The clinical relevance of these findings is supported by a better DFS in patients undergoing NK immunotherapy and depleted of T_regs_. As for the donor, it would be interesting to test whether the composition of the donor NK cell population may be correlated to a different clinical outcome. In particular, the frequency and the function of alloreactive NK cells may impact on the anti-leukemia capacity of infused NK cells. Moreover, the presence within the graft of different subsets of CD56^+^ cells, other than “classic” NK cells, should be evaluated in more detail and, possibly, correlated with the response to NK therapy. The manipulation of donor NK cell graft may represent an interesting approach to increase NK cell activity before infusion. This is a critical point since NK cell-based immunotherapy may be significantly hampered by the transient effector function of infused NK cells. In particular, *in vitro* priming with cytokines, such as IL-12, IL-18, and IL-15, has been reported. This treatment results in the expansion of the memory-like NK cell subset with enhanced functional properties (24) and more prolonged persistence in the host. (25). Recently, in a mouse tumor model a cytokine-based treatment resulted in enhanced anti-tumor activity via the reversal of NK cell anergy, which occurs in the presence of MHC-deficient tumors (26). Since HLA-loss is known to be a fundamental immune escape means for adaptive T-cell-mediated immune response (27), preventing NK-cell anergy may have important clinical implications for cancer immunotherapy.

## Conclusions

Alloreactive NK cells have been emerging as a potent effector cell population against AML. The demonstration of a significant clinical activity of alloreactive purified NK cell infusion outside the transplantation setting represents a proof-of-principle for such an anti-leukemia effect, which has been clearly established in the context of haploidentical SCT. Altogether, these data highlight the pivotal role of NK cells for the development of novel immunological approaches in the clinical management of AML. Nevertheless, several biological issues still require full elucidation. In particular, a more in-depth evaluation of the impact that recipient- and donor-derived factors may have in influencing *in vivo* NK cell activity is an important point. The design of future NK cell-based clinical trials, both in the SCT and adoptive immunotherapy settings, should include a correlation between clinical results and biological outputs. Indeed, correlative biological studies may make it possible to identify those subsets of patients, who may really benefit from NK immunotherapy, in the attempt to tailor both pharmacological and immunological therapies to the patients’ characteristics.

## Conflict of Interest Statement

The authors declare that the research was conducted in the absence of any commercial or financial relationships that could be construed as a potential conflict of interest.

## References

[1] Stern M Passweg JR Meyer-Monard S Esser R Tonn T Soerensen J Pre-emptive immunotherapy with purified natural killer cells after haploidentical SCT: a prospective phase II study in two centers. Bone Marrow Transplant (2013) 48:433–8. 10.1038/bmt.2012.162 22941380

[2] Waldhauer I Steinle A. NK cells and cancer immunosurveillance. Oncogene (2008) 27:5932–43. 10.1038/onc.2008.267 18836474

[3] Borrego F Masilamani M Kabat J Sanni TB Coligan JE. The cell biology of the human natural killer cell CD94/NKG2A inhibitory receptor. Mol Immunol (2005) 42:485–8. 10.1016/j.molimm.2004.07.031 15607803

[4] Campbell KS Hasegawa J. Natural killer cell biology: an update and future directions. J Allergy Clin Immunol (2013) 132:536–44. 10.1016/j.jaci.2013.07.006 23906377 PMC3775709

[5] Curti A Ruggeri L D’Addio A Bontadini A Dan E Motta MR Successful transfer of alloreactive haploidentical KIR ligand-mismatched natural killer cells after infusion in elderly high risk acute myeloid leukemia patients. Blood (2011) 118:3273–9. 10.1182/blood-2011-01-329508 21791425

[6] Terme M Ullrich E Delahaye NF Chaput N Zitvogel L. Natural killer cell-directed therapies: moving from unexpected results to successful strategies. Nat Immunol (2008) 9:486–94. 10.1038/ni1580 18425105

[7] Sutlu T Alici E. Natural killer cell-based immunotherapy in cancer: current insights and future prospects. J Intern Med (2009) 266:154–81. 10.1111/j.1365-2796.2009.02121.x 19614820

[8] Ruggeri L Capanni M Casucci M Volpi I Tosti A Perruccio K Role of natural killer cell alloreactivity in HLA-mismatched hematopoietic stem cell transplantation. Blood (1999) 94:333–9. 10381530

[9] Ruggeri L Capanni M Urbani E Perruccio K Shlomchik WD Tosti A Effectiveness of donor natural killer cell alloreactivity in mismatched hematopoietic transplants. Science (2002) 295:2097–100. 10.1126/science.1068440 11896281

[10] Ruggeri L Mancusi A Capanni M Urbani E Carotti A Aloisi T Donor natural killer cell allorecognition of missing self in haploidentical hematopoietic transplantation for acute myeloid leukemia: challenging its predictive value. Blood (2007) 110:433–40. 10.1182/blood-2006-07-038687 17371948 PMC1896125

[11] Pende D Marcenaro S Falco M Martini S Bernardo ME Montagna D Anti-leukemia activity of alloreactive NK cells in KIR ligand-mismatched haploidentical HSCT for pediatric patients: evaluation of the functional role of activating KIR and redefinition of inhibitory KIR specificity. Blood (2009) 113:3119–29. 10.1182/blood-2008-06-164103 18945967

[12] Passweg JR Tichelli A Meyer-Monard S Heim D Stern M Kühne T Purified donor NK-lymphocyte infusion to consolidate engraftment after haploidentical stem cell transplantation. Leukemia (2004) 18:1835–8. 10.1038/sj.leu.2403524 15457184

[13] Giebel S Locatelli F Lamparelli T Velardi A Davies S Frumento G Survival advantage with KIR ligand incompatibility in hematopoietic stem cell transplantation from unrelated donors. Blood (2003) 102:814–9. 10.1182/blood-2003-01-0091 12689936

[14] Davies SM Ruggieri L DeFor T Wagner JE Weisdorf DJ Miller JS Evaluation of KIR ligand incompatibility in mismatched unrelated donor hematopoietic transplants. Killer immunoglobulin-like receptor. Blood (2002) 100:3825–7. 10.1182/blood-2002-04-1197 12393440

[15] Bornhäuser M Schwerdtfeger R Martin H Frank KH Theuser C Ehninger G Role of KIR ligand incompatibility in hematopoietic stem cell transplantation using unrelated donors. Blood (2004) 103:2860–1. 10.1182/blood-2003-11-3893 15033884

[16] Hsu KC Gooley T Malkki M Pinto-Agnello C Dupont B Bignon JD KIR ligands and prediction of relapse after unrelated donor hematopoietic cell transplantation for hematologic malignancy. Biol Blood Marrow Transplant (2006) 12:828–36. 10.1016/j.bbmt.2006.04.008 16864053

[17] Malmberg KJ Schaffer M Ringdén O Remberger M Ljunggren HG. KIR-ligand mismatch in allogeneic hematopoietic stem cell transplantation. Mol Immunol (2005) 42:531–4. 10.1016/j.molimm.2004.07.037 15607809

[18] Cooley S Weisdorf DJ Guethlein LA Klein JP Wang T Le CT Donor selection for natural killer cell receptor genes leads to superior survival after unrelated transplantation for acute myelogenous leukemia. Blood (2010) 116:2411–9. 10.1182/blood-2010-05-283051 20581313 PMC2953880

[19] Mancusi A Ruggeri L Urbani E Pierini A Massei MS Carotti A Haploidentical hematopoietic transplantation from KIR ligand-mismatched donors with activating KIRs reduces nonrelapse mortality. Blood (2015) 125:3173–82. 10.1182/blood-2014-09-599993 25769621

[20] Meller B Frohn C Brand JM Lauer I Schelper LF von Hof K. Monitoring of a new approach of immunotherapy with allogeneic In-labelled NK cells in patients with renal cell carcinoma. Eur J Nucl Med Mol Imaging (2004) 31:403–7. 10.1007/s00259-003-1398-4 14685783

[21] Miller JS Soignier Y Panoskaltsis-Mortari A McNearney SA Yun GH Fautsch SK Successful adoptive transfer and in vivo expansion of human haploidentical NK cells in patients with cancer. Blood (2005) 105:3051–7. 10.1182/blood-2004-07-2974 15632206

[22] Rubnitz JE Inaba H Ribeiro RC Pounds S Rooney B Bell T NKAML: a pilot study to determine the safety and feasibility of haploidentical natural killer cell transplantation in childhood acute myeloid leukemia. J Clin Oncol (2010) 28:955–9. 10.1200/JCO.2009.24.4590 20085940 PMC2834435

[23] Bachanova V Cooley S Defor TE Verneris MR Zhang B McKenna DH Clearance of acute myeloid leukemia by haploidentical natural killer cells is improved using IL-2 diphtheria toxin fusion protein. Blood (2014) 123:3855–63. 10.1182/blood-2013-10-532531 24719405 PMC4064329

[24] Romee R Schneider SE Leong JW Chase JM Keppel CR Sullivan RP Cytokine activation induces human memory-like NK cells. Blood (2012) 120:4751–60. 10.1182/blood-2012-04-419283 22983442 PMC3520618

[25] Ni J Miller M Stojanovic A Garbi N Cerwenka A. Sustained effector function of IL-12/15/18-preactivated NK cells against established tumors. J Exp Med (2012) 209:2351–65. 10.1084/jem.20120944 23209317 PMC3526364

[26] Ardolino M Azimi CS Iannello A Trevino TN Horan L Zhang L Cytokine therapy reverses NK cell anergy in MHC-deficient tumors. J Clin Invest (2014) 124:4781–94. 10.1172/JCI74337 25329698 PMC4347250

[27] Vago L Perna SK Zanussi M Mazzi B Barlassina C Stanghellini MT Loss of mismatched HLA in leukemia after stem-cell transplantation. N Engl J Med (2009) 361:478–88. 10.1056/NEJMoa0811036 19641204

